# Prognostic Factors for Overall Survival of Patients with Prostate Cancer in Kyadondo County, Uganda

**DOI:** 10.1155/2020/8517130

**Published:** 2020-01-27

**Authors:** James Joseph Yahaya, Tonny Okecha, Michael Odida, Henry Wabinga

**Affiliations:** ^1^Department of Pathology, Makerere College of Health Sciences, Makerere University, P.O. Box 7072, Kampala, Uganda; ^2^Department of Biomedical Science, The University of Dodoma, College of Health Sciences, P.O. Box 395, Dodoma, Tanzania

## Abstract

**Background:**

Prostate cancer is the second most common cancer among men globally. A few studies that have been done in Uganda on survival of patients with prostate cancer indicate that, the overall survival of patients with prostate cancer in Uganda is poor. The aim of this study was to determine the 3-year overall survival rate of a cohort of patients with prostate cancer residing in Kyadondo County who were diagnosed from 2012 to 2014. The secondary objective was to correlate the overall survival with the clinicopathological prognostic factors.

**Materials and Methods:**

This was a retrospective cohort study which involved 136 patients who were diagnosed histologically with prostate cancer at the department of pathology between 2012 and 2014. The cases were registered at the Kampala cancer registry and followed up to 31^st^ December 2017. Data analysis was done using STATA version 12.0. The Kaplan-Meir curves were used for analysis of the 3-year overall survival rate. Hazard ratio (HR) and Log-rank test at 95% confidence interval under Cox-regression model were used to evaluate the effect of the covariates on the 3-year overall survival rate. *p* < 0.05 was considered statistically significant.

**Results:**

More than half of the cases, 55.9% (*n* = 76) had Gleason score >8. Most of the patients, 67.7% (*n* = 92) had advanced disease at diagnosis. The 3-year overall survival rate was 67.6% with median survival of 36.5 months and range of 0–65 months. Clinical stage of the patients (HR = 1.65, *p* = 0.039), Gleason score (HR = 1.88, *p* = 0.008), and lymphovascular invasion (HR = 0.37, *p* = 0.002) were the independent predictors of the 3-year overall survival rate in this study. *Conclusion. * The 3-year overall survival of prostate cancer patients in Uganda is poor. Most of the patients with are diagnosed with advanced clinical stages (stage III and IV). The Gleason score, clinical stage and lymphovascular invasion can powerfully predict independently the overall survival of patients with prostate cancer. This implies that the Gleason score, clinical stage and lymphovascular invasion may be used to predict the overall survival of patients with prostate cancer even prior prostatectomy.

## 1. Introduction

Prostate cancer (PCa) is the leading cause of cancer morbidity and mortality in elderly men worldwide. It is the second and fifth leading cause of cancer-related deaths among men in developing and developed countries respectively [1]. Globally, black men are more affected than whites. Miller et al. [2] reported the incidence rates of PCa for black American men and those from Asia particularly in India, was 228.7 and 141.0 per 100,000 respectively [3]. Uganda is one of the countries in the southern Sub-Saharan Africa (SSA) with very high incidence rate of PCa. In 1990s, PCa in Uganda was among the cancers with highest prevalence [4]. Also in 2011, Uganda was reported to have PCa incidence rate of 38.1 per 100, 000 [5]. This high incidence rate is also reflected by the poor survival of PCa patients in the country. Gondos and associates [5] reported a very low 5-year overall survival rate of patients with PCa of about 49.6%. In a study by Wabinga et al. [6] reported that, the 5-year age-standardized relative survival rates for prostate cancer in Uganda was 46%.

Because of scarcity of follow-up data of cancer patients in developing countries in which Uganda is included, this contributes to shortage of studies involving survival analysis. Moreover, even availability of survival data of patients in Uganda and other countries in the Sub-Saharan Africa where PCa is overwhelming, is still a problem that needs measures to be addressed [7, 8].

The purpose of this study was to determine the 3-year overall survival (OS) rate and also to correlate it with the prognostic factors in patients with PCa diagnosed between January 2012 and December 2014 and followed up to 31^st^ December 2017 in Kyadondo County, Uganda.

## 2. Materials and Methods

### 2.1. Sampling and Patients

This was a retrospective cohort study which involved 136 patients with follow up data who were diagnosed with PCa. The study was conducted in the department of pathology, Makerere College of Health Sciences (MakCHS) in Kampala, Uganda. It involved core needle biopsy materials of patients who were diagnosed histologically with PCa at the department and registered at the Kampala cancer registry (KCR) which is located at the department. KCR is a population-based cancer registry which was established in 1951. It is one of the oldest cancer registries in Africa. It registers cancer patients on different diagnostic basis including clinical, histological, surgical, and autopsy. The registered cancer patients are from Kyadondo County which encompasses the districts of Kampala and Wakiso. The current population is 2, 614, 994 based on the 2014 demographic data. Males and females are 1, 233, 635 and 1, 381, 359 respectively. The cases involved in this study were diagnosed histologically with PCa at the department of pathology between January 2012 and December 2014 and registered at KCR and then followed up for at least 3 years since the date of diagnosis.

### 2.2. Sample Processing

Two formalin fixed paraffin embedded (FFPE) tissue blocks representing left and right lobes of the prostate gland from the patients with PCa were retrieved retrospectively from the repository of the department. Laboratory requisition forms together with the clinical information that was extracted from the database of the cancer registry were used to retrieve the tissue blocks. The retrieved tissue blocks were left to cool at −1.0°C for 10 minutes on the embedding station and then sectioned at 4-micron thickness, deparaffinized on the hot plate at 55°C for 30 minutes, brought down to water and finally stained with standard Harris' hematoxylin stain and counterstained with eosin. Then the prepared slides were reexamined microscopically to confirm the previous histological diagnosis by two independent pathologists who were blinded of the vital status of the patients.

### 2.3. Follow Up of the Patients

We obtained retrospective follow-up data of the cohort of patients with PCa who were registered at KCR and followed up actively for at least 3 years from the day of diagnosis between January 2012 and December 2014. Follow-up time ended on 31^st^ December 2017. Either the patients themselves or their relatives were contacted directly using the phone numbers. Also patients were visited in the wards or at their home. Vital status of every case was recorded in the database. Only cases diagnosed on histology basis were identified for being included in the study provided that the case had complete follow-up data including known vital status at last contact. All selected cases were cross-checked using the selection criteria. The inclusion criteria were: availability of clinical information and tissue blocks as well as complete follow-up data. Cases were excluded for the following reasons: poor biopsy tissue blocks, biopsy material spoilt by insects, and missing clinical data. Every patient that met the inclusion criteria was sampled using nonprobability method.

### 2.4. Data Collection and Analysis

Data collected were double cross-checked and edited for every existing error and analysis was performed using STATA version 12.0. Continuous variables were presented in the form of mean (SD), median, and range whereas categorical variables were presented in proportions. Kaplan–Meier (K–M) curves were used to analyze the 3-year OS rate of the cases. All cases without the event of interest (death) at the end of the follow-up period were censored. Patients who were lost to follow up were censored as well. Univariate and multivariate analysis using Cox regression model was applied. Hazard ratio (HR) and Log rank tests were used to study the effect of the independent variables on the 3-year OS rate at 95% confidence interval (CI). A two-tailed *p* < 0.05 was considered statistically significant.

## 3. Results

The mean (SD) age of the patients was 69.0 (SD = 10.37) years with range of 45–99 years. The peak age of the patients in this study was 60–69 years. The median prostate specific antigen (PSA) was 96.8 ng/mL with range of 2.7–2000.0 ng/mL. Most of the patients, 55.1% (*n* = 75) had PSA greater than 20.0 ng/mL. Patients with PSA ≤10.0 ng/mL were 11.0% (*n* = 15), and only, 1.5% (*n* = 2) of the cases had PSA ≤4.0 ng/mL. Clinical staging among the patients was done by physical examination, digital rectal examination (DRE), and transrectal ultrasonography (TRUS). Other patients were staged by means of computed tomography (CT) scan and magnetic resonance image (MRI).

Table 1 represents the different clinical characteristics of the patients in the study. Most of the patients had advanced PCa, consisting of 67.7% (*n* = 92) clinical stage III and IV. The mean tumour extent (TE) was 60.1% (*SD* = 17.27) with a range of 25.0–100.0%. Most of the cases, 41.2% (*n* = 56) had involvement of the tumour from 51.0–75.0% of the sampled needle cores followed by 36.0% (n = 49) with involvement of 26.0–50.0%. GS≥9/Grade Grouping (GG) 5 was the dominating tumour grade which comprised of 36% (*n* = 49) followed by GS≤6/GG 1 which constituted 21.3% (*n* = 29). Other grades are as seen in Table 1. Lymphovascular invasion (LVI) and perineural invasion (PNI) were present in 29.4% (*n* = 40) and 42.7% (*n* = 58) respectively.

**Figure 1:**
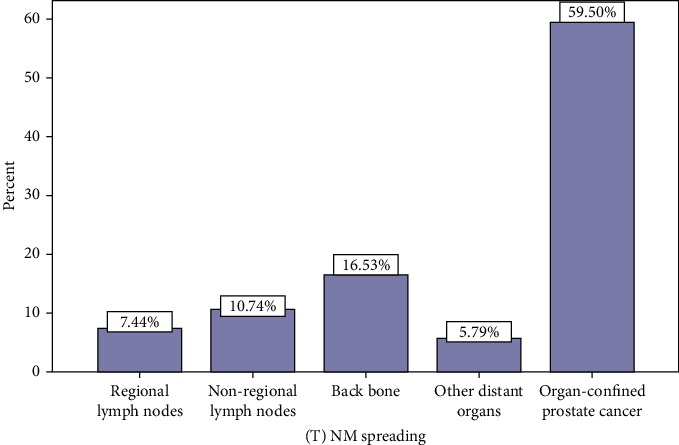
Spreading of prostate cancer in the patients. Back bone was the most distant organ involved by the spreading cancer.

Regarding the 40.5% (*n* = 49) of patients with lymph node involvement and metastasis to distant organs, it was found that, 18.2% (*n* = 22) had lymph node involvement and 22.3% (*n* = 27) had involvement of distant organs other than lymph nodes (Figure 1).

The types of treatment provided to our patients are shown in Table 2. Androgen deprivation therapy (ADT) were provided medically and surgically for 31% (*n* = 42) and 22% (*n* = 30) patients, respectively. Docetaxcel was provided for 14% (*n* = 19) of the cases.

When the 3 years of follow-up ended, 29.4% of the patients were lost to follow-up, making the follow-up rate in this cohort to be 70.6%. The 3-year OS rate for the patients was 67.6% (*n* = 92). The median period of survival was 36.5 months with a range of 0–65 months.

Table 3 shows both the clinical prognostic factors (age, PSA, and clinical stage) and pathologic prognostic factors obtained from the needle core biopsies (TE, GS, LVI, and PNI) which were tested using univariate and multivariate analyses for their predictive value of the 3-year OS rate. Statistically significant predictors at diagnosis for overall survival in the univariate analysis were GS (*p* = 0.003), PNI (*p* = 0.001), pretreatment PSA (*p* = 0.007), clinical stage (*p* = 0.006), and LVI (*p* = 0.0005). Patients with pretreatment PSA >20 ng/mL were 2.96 times more likely to die compared to the ones with PSA ≤20 ng/mL and the two compared groups were statistically significantly different (*p* = 0.007). Patients with advanced PCa (stage III and IV) were 3.34 times more likely to die than those with prostate-confined cancer (stage I and II) and the difference was statistically significant (*p* = 0.006). The HR of 2.54 for patients with GS of ≥8 for example, means that at any point during follow-up these patients have a 2.5 fold increased risk of dying, compared with patients of GS <8. Patients with PNI were 63% more likely to die within the 3 years of follow-up compared to those without PNI and the difference was statistically significant (*p* = 0.001).

LVI which is a well-known pathologic prognostic factor for predicting the OS rate of patients with PCa, under univariate analysis using Cox regression model in this study it was found to correlate with the 3-year OS rate (*p* = 0.0005). Patients who were found to have no LVI, their probability of dying within the follow up period of 3 years was 75% less likely than those with LVI. The TE variable did not predict the 3-year OS rate of the patients in this study even in univariate analysis although patients with TE >50% were 1.67 times more likely to die compared to those with maximum core biopsy involvement (TE) ≤50%. Likewise, age did not predict the 3-year OS rate under univariate analysis despite the fact that patients who were aged >60 years were 31% more likely to die within the follow-up period compared to the patients aged ≤60 years and the difference was not statistically significant (*p* = 0.216).

In the multivariate analysis, GS (*p* = 0.008), LVI (*p* = 0.002), and clinical stage (*p* = 0.039) remained statistically significant predictors for the OS rate (Table 3).

**Figure 2:**
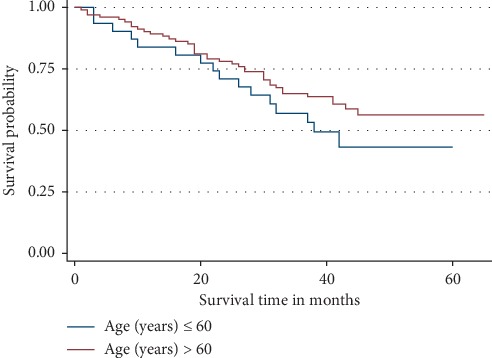
Overall survival by age group of the patients. Patients aged more than 60 years show a relatively increased risk of dying compared to those aged less than 60 years.

In this study, although the risk of dying among patients with age >60 years, the 3-year OS rate was not significantly different from the patients who had age <60 years (*p* = 0.581) although the ones aged >60 years were 17% more likely to die than those aged <60 years. Even the K-M curves for the two age groups compared were almost overlying (Figure 2).

**Figure 3:**
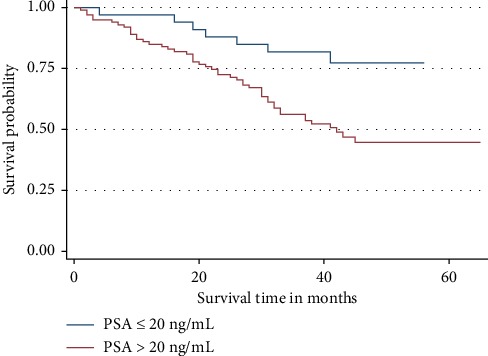
Overall survival by PSA. Patients with PSA >20 ng/ml were dying more than those with PSA ≤20 ng/mL.

The probability of dying among patients with PSA >20 ng/mL in our study was higher than those with PSA ≤20 ng/mL. This contributed to the gap between the 3-year OS rate between the two groups although the difference of the survival rate was not significantly different (*p* = 0.346) (Figure 3).

**Figure 4:**
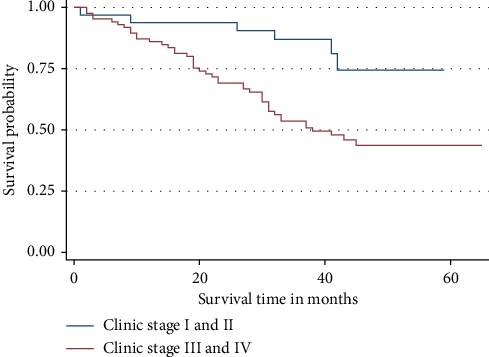
Overall survival by clinical stage. The risk of dying was increasing with progression of the disease.

The risk of dying among the patients included in this study was increasing with increase in the clinical stage. Patients with organ-confined PCa (stage I and II) were dying at a long interval compared to those with advanced PCa. However, at the beginning of the follow-up period, the rate of dying between the two groups was almost similar (Figure 4). Additionally, it was also observed that, the difference in the 3-year OS rate between the two groups was statistically significant (*p* = 0.03).

**Figure 5:**
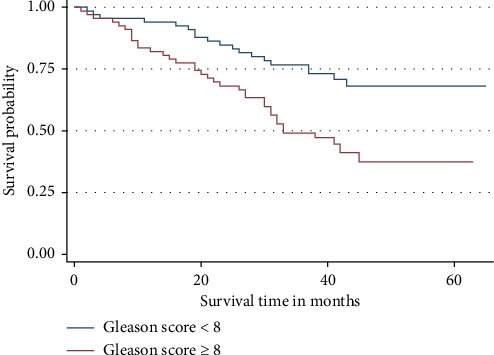
Overall survival by Gleason score. Patients with poorly differentiated disease were more likely to die than the ones with differentiated disease.

Figure 5 shows the K–M curves for GS in relation to 3-year OS rate. The correlation of GS and the 3-year OS rate was highly statistically significant (*p* = 0.008). Patients in the group with GS ≥8 at any time point during the follow up period were 1.88 times more likely to die than patients in the group with GS <8. Below 10 months of survival after diagnosis, patients in both groups had almost equal survival. Thereafter, those with GS ≥8 their OS rate decreased compared to those with GS <8.

**Figure 6:**
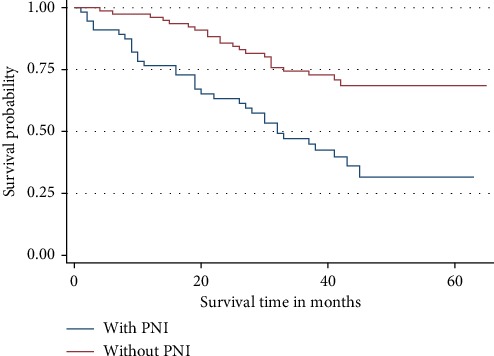
Overall survival by perineural invasion (PNI). Patients with PNI had a high tendency of dying than those without PNI, although the difference was not statistically significant.

Figure 6 represents the K–M curves for PNI and how it correlated with the 3-year OS rate. PNI was not an independent predictor of the 3-year OS rate under multivariate analysis as it was in univariate analysis even though from the curves it can be observed that patients with PNI had lower OS rate compared to those without PNI. Patients without PNI were 37% less likely to die. However, the difference was not statistically significant (*p* = 0.176).

**Figure 7:**
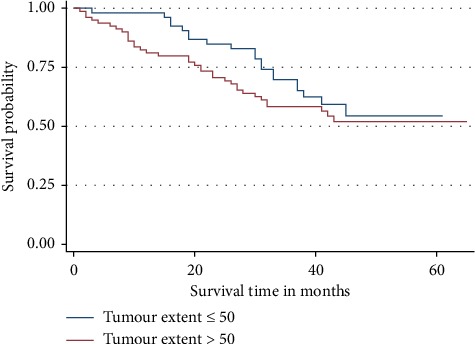
Overall survival by tumour extent (TE). Patients with high TE were dying more than those with low TE.

Patients with TE > 50% were 8% times more likely to experience the event of interest (death) during the study period compared to the cases with TE ≤50% but the difference was not statistically significant (*p* = 0.801) (Figure 7).

**Figure 8:**
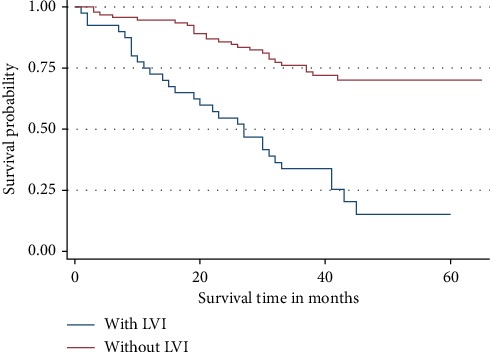
Overall survival by lymphovascular invasion (LVI). LVI was predicting the overall survival independently.

When patients with LVI and those without LVI were compared for the 3-year OS, it was found that those without LVI were 63% less likely to die within the 3-year period of the follow up than those with LVI and the difference was statistically significant (*p* = 0.002) (Figure 8).

## 4. Discussion

The overall survival of cancer patients including PCa is always subject to a number of prognostic factors that influence the outcome of the patients. The Clinical stage of the disease and the grade of the disease are of paramount importance in prognostication. The information on the survival of cancer patients in the Sub-Saharan Africa (SSA) is very scarce in spite of high prevalence and mortality across the region [9]. Shortage of sufficient cancer registries and lack of comprehensive follow-up of patients with cancer including those with PCa in the SSA region remains a major obstacle for availability of survival analysis among cancer patients.

For example, in Uganda, there are limited number of studies involving survival analysis of any type for different types of cancers in spite of having a very old cancer registry in place. Three previous studies done on the OS rate of patients with PCa in Uganda include that of Gondos et al. [5] in 2005 which involved cases registered at the KCR. This study reported that, the 5-year OS rate of patients with PCa in Kyadondo County was 49.6%. Another study is that of Okuku et al. [10] which involved cases that were admitted at the Uganda cancer institute (UCI) and it reported that, the 1-year OS rate of the patients was 75.0%. In 2014, Wabinga et al. [4] reported only 46% for the 5-year OS rate among patients with PCa. All the three studies did not include correlation of prognostic factors unlike for this study which included correlation of prognostic factors with OS rate.

Patients with PCa in developed countries have better OS rate than the ones in developing countries [11–16]. In 2017 Ukawa et al. reported a tremendous 3-year OS rate of 88.6% in patients with PCa in the BioBank Japan Project which included 4793 cases [17]. This is higher than 67.6% for the 3-year OS rate of the patients in this series. The most likely reason for this big difference in survival between the two settings is that, in developed countries, PCa is detected earlier than in the developing countries. This helps to improve prognosis. Other reasons include good socio-economic status, better treatment modalities for PCa patients in Japan and genetical predispositon for poor prognosis of patients with PCa in the African population. 

In 2014, Rugwizangoga et al. [18] from the neighbouring country of Tanzania reported 54.7% of 5-year OS rate among the cases with PCa. Pinkawa reported a 69.0% of 3-year OS rate among the patients with PCa from the central south part of China which is almost similar to 67.6% reported in the current series [19]. Studies have shown that patients with PCa in most of areas in China are diagnosed with advanced clinical stage similar to the patients in the SSA in which Uganda is included. This makes them to have a similar survival time frame.

GS, LVI, and clinical stage were the prognostic factors which predicted independently the 3-year OS rate of the patients in this study. Age of the patients, pre-treatment -PSA, TE, and PNI did not predict independently the OS rate when they were subjected to multivariate analysis, although some of them (age of the patients and PSA) showed correlation under univariate analysis. GS has been reported to have the ability of predicting independently the OS rate of PCa patients in many studies. For instance, Rugwizangoga and colleagues [18] reported that there was a correlation between GS and 5-year OS rate of the patients with PCa (*p* = 0.021). Angwafo et al. [20] also reported a correlation of GS with OS rate among the patients with PCa in Cameroon. The presentation of patients with PCa in Uganda with high GS as it was reported by Yahaya [21] is an indication that most of them end up having poor clinical outcome.

### 4.1. Correlation of Age of the Patients with 3-Year Overall Survival of the Patients

Age was not a predictor of the 3-year OS rate in this study similar to the finding in the study by Ekeke et al. in Nigeria [22]. The similarity for the lack of correlation between the two studies could be explained by the fact that, in both studies most of the patients were in 60 s years of age. It seems that patients with PCa in their 60 s have at least better performance than those in their 80 s. This is different from other studies which reported that age was the predictor of the OS rate [23–26]. The possible reason for the lack of correlation between age and OS rate in our study is that, majority of the patients in this study were in the 60 years of age unlike in other studies that have reported a correlation between the two variables. For example, Kan et al. reported that, patients aged >80 years had poor 5-year OS rate compared to patients who were aged ≤80 years (*p* < 0.001) [24]. Increased age is usually associated with poor performance of the patients which in turn is more likely to increase mortality of the patients [25, 27].

### 4.2. Correlation of Clinical Stage of the Patients with 3-Year Overall Survival of the Patients

Clinical stage has been reported to be one of the potential clinical predictors of survival in patients with cancer including PCa. In this study clinical stage was one of the predictors of the 3-year OS rate. This is similar to the observation in both developed and developing countries. In Tanzania, Rugwizangoga et al. reported that there was a positive correlation between the clinical stage and the 5-year OS rate in patients with PCa (*p* = 0.018). In a study by Xu et al. [28] which was done in China, it was reported that, the 5-year OS rate was decreasing with increase in the clinical stages of the patients with PCa and the difference was statistically significant (*p* = 0.0001).

### 4.3. Correlation of Prostate-Specific Antigen (PSA) with 3-Year Overall Survival of the Patients

Studies have shown that in places where there is mass screening for PCa, pretreatment PSA plays a role as a predictor for both OS rate and prostate-specific survival (PSS) [25, 28]. Additionally, Kan et al. reported that PSA reduction >90% of the pretreatment PSA was associated with better OS rate of the patients with PCa [24]. However, in settings where screening for PCa is not a common practice, the ability of pretreatment PSA to predict the OS rate in patients with PCa seems to be different from the situation in developed countries. For instance, Ekeke et al. in Nigeria reported that, Pretreatment PSA was not associated with 5-year OS rate (*p* = 0.384) [22]. This finding is in keeping with our finding in this study whereby the correlation between pre-treatment PSA and the 3-year OS rate was not statistically significant (*p* = 0.346).

### 4.4. Correlation of Gleason Score (GS) with 3-Year Overall Survival of the Patients

Gleason Score (GS) remains one of the pathologic prognostic markers that can predict independently the OS of PCa patients with a very high reproducibility of results among different studies unlike other prognostic factors [12, 29]. In countries where patients with PCa are diagnosed with low GS, the OS among the patients has been found to be very high. Parra et al. in Spain reported that GS 8 was the main score with the ability to predict independently the OS of the patients with a statistical significance (*p* = 0.006) [30].

Galego et al. in Portugal reported a correlation between 5-year OS and GS (*p* < 0.05) [31]. In their study they also found that GS 7 was an independent predictor of the biochemical relapse of the disease unlike PSA. Early detection of PCa is usually linked with low stage at diagnosis as well as low tumour grade [31, 32]. For countries where screening of PCa is massive and sustainable, it has been found that most of the patients are diagnosed at early stage and for that matter they have low tumour grade which is usually determined by GS [33]. This in turn helps them to have better clinical outcome and therefore, survive longer.

### 4.5. Correlation of Lymphovascular Invasion (LVI) with 3-Year Overall Survival of the Patients

Lymphovascular invasion (LVI) in this study predicted the 3-year OS of the patients independently unlike PNI. The LVI plays a role of being the sine qua non for spreading of cancers including PCa. Although reports have shown that the role of PVI in predicting the OS in patients with PCa including biochemical progression free-survival (BCPFS) is debatable, however, there are quite a number of studies in the literature which support that the LVI is an independent predictor of survival in PCa including OS rate as it was the case in this study [30, 34]. Baydar and associates in Turkey reported that LVI predicted both the BCPFS and the 3-year OS rate in both univariate and multivariate analyses (*p* = 0.023) and (*p* = 0.001) respectively which is in keeping with the findings in the present study [34]. Studies have also clearly shown that LVI is associated with other adverse clinicopathological characteristics of patients with PCa [12, 18, 34].

### 4.6. Correlation of Perineural Invasion (PNI) with 3-Year Overall Survival of the Patients

Perineural invasion (PNI) has become a relevant and potential pathologic prognostic factor, in spite of being not extensively investigated among the aspects of tumor biology in most of the malignancies, including PCa. The role of PNI and its prognostic role to clinicians remains a debatable subject [19, 29]. PNI has been found to correlate with most of the adverse clinicopathological characteristics among the patients with PCa. Studies have shown that patients with PNI detected from pretreatment specimens (needle core biopsies) have a high chance of being found with positive surgical margin (PSM), seminal vesicle involvement (SVI), high GS, advanced stage, lymph node involvement (LNI), and extraprostatic extension (EPE) [13, 18, 35, 36].

Lee JT and associates [37] found that PNI was predicting the 5-year OS rate of patients with PCa by univariate analysis (*p* = 0.023), however, in multivariate analysis, PNI was not an independent prognostic factor of the 5-year OS rate (*p* = 0.726). This finding is in agreement with the finding in this study whereby PNI was predicting the 3-year OS rate of the patients in univariate analysis (*p* = 0.001) and not in multivariate analysis (*p* = 0.329). PNI apart from being a predictor of OS rate at some point, it has also been reported to predict biochemical progression-free survival among patients with PCa regardless of the type of specimens used for evaluation.

### 4.7. Correlation of Tumour Extent (TE) with 3-Year Overall Survival of the Patients

Tumour extent (TE) determination currently has gained value in the prognosis analysis of cancer patients including those with PCa. The existence of variation in the methodology used to determine TE in the needle core biopsies which includes difference in sampling areas of the needle core biopsies, lack of universal standards in setting the cut off points and variation in the way of determining the maximum areas with tumour involvement, all these have compromised the clinical utility of TE in using it as a histopathologic prognostic marker for pretreatment prediction of the presence of adverse prognostic factors [38]. Because of the lack of universal standards in tumour quantification using needle core biopsies, this has brought about disagreement across different studies. In the work of Rugwizangoga et al. [18] who used the same methodology as the one used in the current study, it was reported that, TE was increasing with GS and the association was statistically significant (*p* = 0.0003) similar to what was found in this study (*p* = 0.001).

The current study used the percentage of positive needle core biopsies which was obtained by dividing the number of positive needle core biopsies to the total number of needle core biopsies sampled. Then the ratio obtained was multiplied by 100%. This method is similar to the method that was used in the study of Huang et al. in which they used a cut-off value of >50% positive biopsy cores [13]. They found that the percentage of positive biopsy cores is a strong and independent predictor for metastasis-free and overall survival (*p* < 0.05). This is not in line with what was found in this study. TE was not the predictor of the 3-year OS rate for either univariate (*p* = 0.103) or multivariate analysis (*p* = 0.389) despite the similarity in methodology that was used.

The difference in sextant sampling and also optimal number of needle core biopsies for every case between the two studies might have resulted in the difference of the findings. Besides, since the methodology in the two studies involves visual and manual estimation of the percentage of the area involved by tumour cells in the needle core biopsies, this might have contributed to interobserver difference between the two series. In another study in which the percentage of positive biopsy cores was adjusted for the percentage of cancer in needle-biopsies, TE did not have prediction for the BCPFS (*p* = 0.09) and OS rate (*p* = 0.081) [39]. The use of needle core biopsies in determining prognosis of PCa during pretreatment is of advantage in many different ways including predicting the survival of the patients before treatment.

## 5. Conclusion

Majority of patients with PCa in Uganda present with very high PSA, GS, and advanced clinical stage at diagnosis. Because of presenting with very high tumour grade and advanced clinical stage, this contributes to most of them to end up with poor clinical outcomes despite that there are modern diagnostic tests as well as improved treatment options in the country. The 3-year OS rate of the patients in this study was as lower as what has been reported elsewhere in the developing countries, especially in the SSA region. The prognostic factors correlated with the 3-year OS rate for both univariate and multivariate analysis in less similar way as what has been reported in other studies. The GS, clinical stage and LVI in this study predicted the 3-year OS rate independently for both univariate and multivariate analysis unlike PNI and TE which were not able to predict the 3-year OS rate independently.

## Figures and Tables

**Table 1:** Clinical and pathological characteristics of the patients (*n* = 136).

Characteristic		No of cases (*N*)	Percentage (%)
Age group (years)	≤50	10	7.4
	>50	126	92.6
PSA (ng/ml)	≤10	15	11.0
	11–20	20	14.7
	>20	75	55.1
	Missing	26	19.1
Clinical stages	I	5	3.7
	II	24	17.6
	III	56	42,2
	IV	36	26.5
	Missing	15	11.0
Tumour extent	<50%	54	39.7
	≥50%	82	60.3
GS	3 + 2 = 5	5	3.7
	3 + 3 = 6	24	17.6
	3 + 4 = 7	13	9.6
	4 + 3 = 7	18	13.2
	4 + 4 = 8	27	19.9
	9	34	25
	5 + 5 = 10	15	11
GG	GG 1: ≤6	29	21.3
	GG 2: 3 + 4 = 7	13	9.6
	GG 3: 4 + 3 = 7	18	13.2
	GG 4: 4 + 4 = 8	27	19.9
	GG 5: 9-10	49	36
Perineural invasion	Present	58	42.7
	Absent	78	57.3
Lymphovascular invasion	Present	40	29.4
	Absent	96	70.6

GS: Gleason score, GG: grade grouping, PSA: prostatic-specific antigen.

**Table 2:** Different treatment approaches of the patients.

Treatment	No. of patients (N)	Percentage (%)
Luteinizing hormone-releasing hormone agonist	42	31
Orchiectomy	30	22
Prostatectomy	57	42
Radiotherapy	39	28.7
Docetaxel	19	14
Bisphosphonate	53	39

Note: Some patients received more than one treatment.

**Table 3:** Univariate and multivariate cox regression analysis for 3-year overall survival rate.

Variable		Univariate analysis		Multivariate analysis	
		HR(95% CI)	*p*	HR(95% CI)	*p*
Age (years)	≤60	1	0.216	1	0.581
	>60	0.69 (0.39–1.24)		0.83 (0.43–1.60)	
Pre-treatment PSA (ng/mL)	≤20	1	0.007	1	0.346
	>20	2.96 (1.34–6.57)		1.05 (0.54–5.66)	
Clinical stage	I and II	1	0.006	1	0.039
	III and IV	3.34 (1.42–7.84)		2.65 (1.78–4.19)	
GS	<8	1	0.003	1	0.008
	≥8	2.54 (1.37–4.70)		1.88 (3–7.11)	
TE	≤50%	1	0.103	1	0.801
	>50%	1.67 (0.90–3.08)		0.92 (0.49–1.71)	
PNI	No	1	0.001	1	0.176
	Yes	0.37 (0.21–0.67)		0.63 (0.32–1.23)	
LVI	No	1	0.0005	1	0.002
	Yes	0.25 (0.14–0.45)		0.37 (0.18–0.69)	

GS: Gleason score, PSA: prostatic specific antigen, TE: tumour extent, PNI: perineural invasion, LVI: lymphovascular invasion, CI: confidence interval, HR: hazard ratio. Reference HR = 1.

## Data Availability

The datasets generated and/or analyzed during the current study are not publicly available due to the reason that they are restricted from being shared publicly but are available from the corresponding author on request.
